# Elderly Patients with Nondistant Metastatic Pancreatic Head Adenocarcinoma Cannot Benefit from More Radical Surgery

**DOI:** 10.1155/2022/6469740

**Published:** 2022-04-18

**Authors:** Li Chen, Lanning Jia, Zhigang Tian, Yang Yang, Ke Zhao

**Affiliations:** ^1^Department of General Surgery, Tianjin Medical University General Hospital, Tianjin Medical University, Tianjin, China; ^2^Department of Anorectal Surgery, Anorectal Surgery Center, Union's Hospital of Tianjin, Tianjin, China

## Abstract

**Background:**

The incidence of pancreatic cancer continues to rise globally, with pancreatic head cancer accounting for nearly 60–70%. Pancreatic head cancer occurs mainly in people over the age of 60, and its morbidity and mortality increase with age. We investigated whether these elderly patients with nondistant metastases would benefit more from expanded pancreaticoduodenectomy (EPD) compared with standard pancreaticoduodenectomy (SPD).

**Methods:**

3317 elderly patients with pancreatic head cancer from the SEER database were included in the study based on the inclusion and exclusion criteria. These patients were divided into a nonsurgical group and surgical group (including EPD and SPD). Univariate and multivariate Cox proportional hazards models were applied to identify the independent risk factors for cancer-specific survival (CSS). The survival differences between the nonsurgical group and surgical group were compared. Propensity score matching (PSM) methods were applied to balance covariates and reduce the interference of confounding variables. The two groups of patients were matched in a 1 : 1 ratio, and the covariates between the two groups were compared to verify the matching validity. The survival difference in different groups was compared after the matching analysis.

**Results:**

3317 enrolled patients were divided into the surgical group (*n* = 984) and nonsurgical group (*n* = 2333). Before PSM, there were significant differences in overall survival (OS) and CSS between the nonsurgical group and surgical group (median OS: 8 months vs. 20 months, *P* < 0.001; median CSS: 8 months vs. 22 months, *P* < 0.001). The multivariate CSS Cox regression analysis demonstrated surgery is an independent risk factor. However, no significant differences were founded between the SPD and EPD groups (median OS: 20 months vs. 22 months, *P*=0.636; median CSS: 22 months vs. 22 months, *P*=0.270). After PSM, there were also no significant differences in OS and CSS between the SPD and EPD groups (median OS: 23 months vs. 18 months, *P*=0.415; median CSS: 26 months vs. 18 months, *P*=0.329).

**Conclusion:**

This study uses PSM to evaluate the effects of EPD and SPD for elderly patients with nondistant metastatic pancreatic head adenocarcinoma. It found that surgery is an independent prognostic factor, but expanded surgery has no survival advantage for these patients, whereas SPD provides a better survival advantage than EPD. SPD is a reasonable treatment option for these patients.

## 1. Introduction

The incidence of pancreatic cancer continues to rise globally. Statistics from the United States in 2021 showed that the incidence of pancreatic cancer ranks 10th among males and 9th among females. It is the 4th cause of cancer-related mortality after lung, prostate (male), breast (female), and colon cancer and is expected to be the second cause by 2030 [1, 2]. The anatomy of the pancreas is divided into head, body, and tail. Pancreatic head cancer accounts for nearly 60–70% [3, 4]. Adenocarcinoma accounts for more than 90% of all pathological types of pancreatic malignancies [5]. Pancreatic cancer occurs mostly in people over 60 years old, and its morbidity and mortality increase with age [6, 7]. It is foreseeable that its incidence will increase with the accelerating aging process. Elderly patients generally have a worse prognosis than younger patients due to their poor conditions and comorbidities [8, 9]. Even for those with good physical conditions, factors such as age and postoperative complications may prevent them from receiving more radical treatments [10].

The treatment options and efficacy have improved in recent years. Surgery, chemotherapy, and drug-targeted therapy have been proved to be beneficial for survival. However, the average 5-year survival rates are still less than 10% [1, 11]. Primary tumors of the pancreatic head often invade surrounding blood vessels, especially the portal vein (PV) and superior mesenteric vein (SMV) due to their biological characteristics and anatomical particularities [12], which may relate to a poor prognosis. Currently, surgery is the only effective treatment that may cure pancreatic cancer [11, 13, 14]. However, since most patients are already at an advanced stage when they are first diagnosed, only 20% of patients have the surgery opportunity [15]. With the development of surgical technology, the postoperative mortality in some experienced centers has dropped to 1%, but the 5-year survival rate after surgery is still not optimistic [16]. For patients diagnosed with locally resectable lesions, the 5-year survival rate after surgery is only 20% [17]. A population-based study in the United States showed that the postoperative 5-year survival rate for clinical stage I patients was 24.6% [18]. Even for most patients who received radical resection, their 5-year survival rate is less than 25% [11].

The classic surgical treatment for pancreatic head cancer is standard pancreaticoduodenectomy (SPD), also known as Whipple surgery [12]. Some scholars believe that the Whipple procedure is difficult to achieve radical resection, and expanded pancreaticoduodenectomy (EPD) should be prioritized. The International Study Group for Pancreatic Surgery (ISGPS) defined the scope of extended resection, which mainly includes adjacent invaded blood vessels and organs, not involving expanded lymph node dissection [19].

However, which surgical method can provide a better survival advantage is still controversial, especially for these elderly patients, and whether they would benefit more from radical surgery than less invasive operation has not been determined. Currently, there are few reliable large-scale studies of pancreatic head cancer patients over 60 years of age without distant metastasis.

Surveillance, Epidemiology, and End Results (SEER) is a publicly available oncology database that collects information of nearly 30% of the US population [20]. In this work, we collected the data of pancreatic head cancer patients aged over 60 without distant metastases (stage I–III, AJCC 8th edition) in the SEER database to determine whether more radical surgery should be recommended and explore the survival risk factors.

## 2. Materials and Methods

### 2.1. Data Source

Data were collected from SEER^*∗*^Stat software, version 8.3.9.2, of the National Cancer Institute. According to the third edition of the International Classification of Oncological Diseases (ICD-0-3), the code for ductal adenocarcinoma is 8140/3. The following information was collected: pathological type, primary site, marital status, age, race, sex, diagnosis year, grade, tumor size, T stage, N stage, M stage, regional positive nodes, surgery method, radiation, chemotherapy, survival time, overall survival (OS) and cancer-specific survival (CSS) outcome, etc. The inclusion criteria were as follows: (1) pathologically proven pancreatic cancer (histological code 8140/3, adenocarcinoma, NOS), (2) primary lesions located in the pancreatic head (site code C25.0, pancreatic head), (3) diagnosed from January 1, 2004, to December 31, 2015, (4) age 60 years or older, and (5) no distant metastasis. The following exclusion criteria were adopted: (1) samples with missing or incomplete clinical information, (2) no detailed surgical methods records, and (3) survival less than 1 month. The 8th edition of the clinical AJCC TNM staging manual was applied in this study. The SEER database did not require ethical approval from official agencies. We signed a usage agreement form to access the data file, and the reference number is 15873-Nov2020.

### 2.2. Study Design

Since the SEER provides relatively complete clinical data and outcome events, a clinical retrospective study was used to explore the effect of different surgical methods on the prognosis of patients. Firstly, the enrolled patients were included in cohort 1 based on the above inclusion and exclusion criteria and divided into the nonsurgical group and surgical group. We compared the differences in baseline data between the two groups. Subsequently, the independent risk factors for CSS in those patients were analyzed to determine whether surgery is one of them. We further integrated the original surgical variables and divided them into SPD and EPD and compared survival differences between the groups. Propensity score matching (PSM) is a statistical method that effectively reduces the confounding bias of observational studies and makes them similar to randomized controlled studies [21]. Surgery was used as the grouping variable to match the patients at a ratio of 1 : 1. Match tolerance was set to 0.02, which makes the matching covariates more balanced. Cohort 2 was formed after matching analysis. Then, the baseline data of the groups were compared, as well as the difference in survival between the groups.

### 2.3. Statistical Analysis

The baseline data of the nonsurgical group and the surgical group were compared by the *t*-test or *χ*^2^ test. X-tile software was used to find the best cutoff values for age and tumor size, which were prepared for subsequent Cox analysis. Univariate and multivariate Cox proportional hazards models were applied to identify the independent risk factors for CSS in cohort 1. The Kaplan–Meier survival curve was used to determine the OS and CSS rates, the log-rank test was used for assessing the difference, and the average survival time of different groups was recorded. PSM was used to balance covariates for match patients. The forest plot of HR is used to assess the intensity of the association between potential prognostic factors and CSS. *P* < 0.05 was considered statistically significant, and the hazard ratios with 95% confidence interval (CI) are also recorded. All statistical analyses were conducted by Statistical Products and Services Solutions software (SPSS, version 26.0) and R software (version^*∗*^ 64 4.1.1).

## 3. Results

### 3.1. Clinical Features

A total of 3317 eligible patients were included from the SEER database. 984 patients received the above-mentioned two surgical methods, and 2333 patients were in the nonsurgical group. The baseline characteristics before PSM is summarized in Table 1. The age of the surgical group was slightly younger than that of the nonsurgical group, 70.61 (±6.67) and 75.55 (±8.70), respectively, *P* < 0.001. The tumor size in the surgery group was slightly smaller than that in the nonsurgical group, 31.96 (±13.87) and 35.13 (±17.79), respectively, *P* < 0.001. The statistical differences also can be seen in the other 9 categorical variables. After PSM, the distribution of all variables for the 704 patients (352 pairs) became more balanced, and baseline characteristics can be found in Table 2.

### 3.2. Univariate and Multivariate Cox Regression Analysis

X-tile software was used to find out the optimal cutoff for age and tumor size. The optimal cutoff values for age were 73 and 82 years; and the optimal cutoff values of tumor size were 26 and 41 mm (Supplementary Figure 1). Some risk factors were closely related to CSS probability in the univariate and multivariate Cox proportional hazard ratio models, as shown in Table 3. For example, surgery (HR: 0.338; 95% CI: 0.296–0.386; *P* < 0.001) and tumor size (HR: 1.261; 95% CI: 1.138–1.299; *P* < 0.001). In addition, age (HR = 1.148; 95% CI: 1.087–1.211; *P* < 0.001), N stage (HR = 1.281; 95% CI: 1.180–1.390; *P* < 0.001), radiotherapy (HR = 0.863; 95% CI: 0.794–0.939; *P*=0.001), and chemotherapy (HR = 0.555; 95% CI: 0.508–0.607; *P* < 0.001) were also independent risk factors of CSS in pancreatic head adenocarcinoma patients over 60 years old.

### 3.3. Survival Analysis

**Figure 1:**
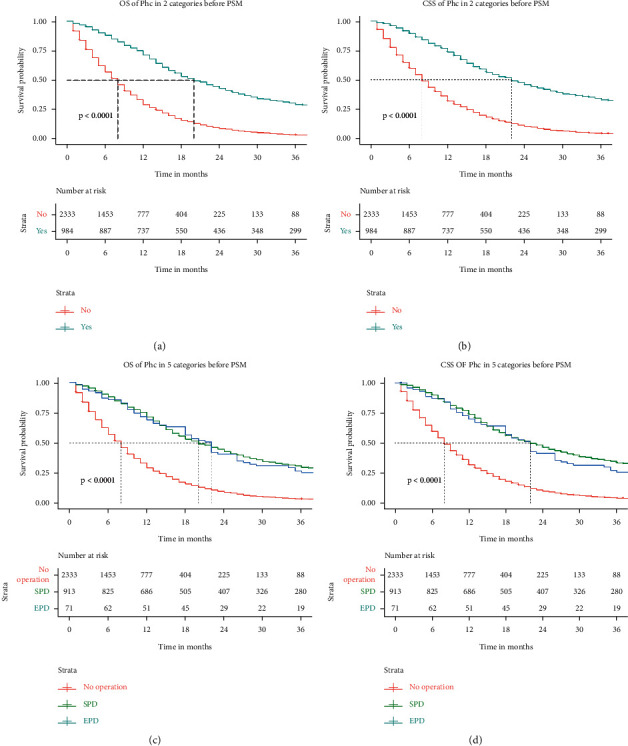
OS (overall survival) and CSS (cancer-specific survival) analysis of pancreatic head cancer patients before PSM (propensity score matching). SPD: standard pancreaticoduodenectomy; EPD: expanded pancreaticoduodenectomy.

**Figure 2:**
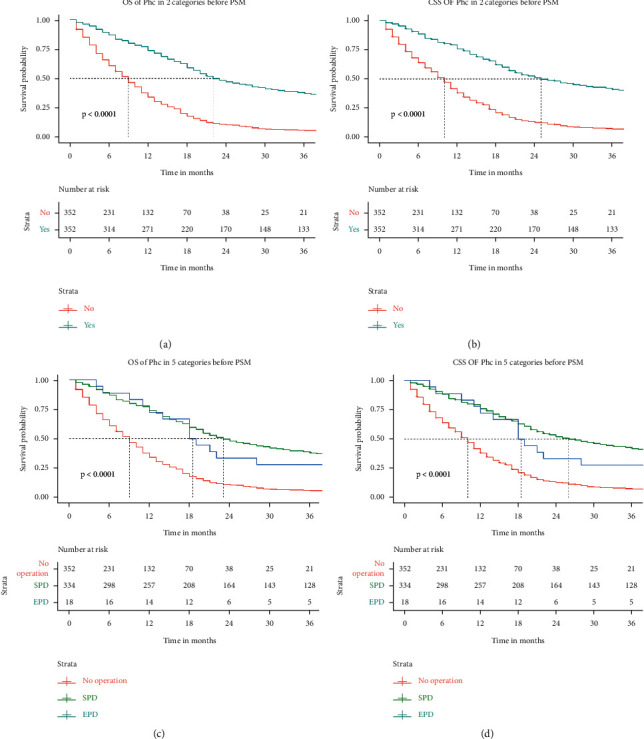
OS (overall survival) and CSS (cancer-specific survival) analysis of pancreatic head cancer patients after PSM (propensity score matching). SPD: standard pancreaticoduodenectomy; EPD: expanded pancreaticoduodenectomy.

**Figure 3:**
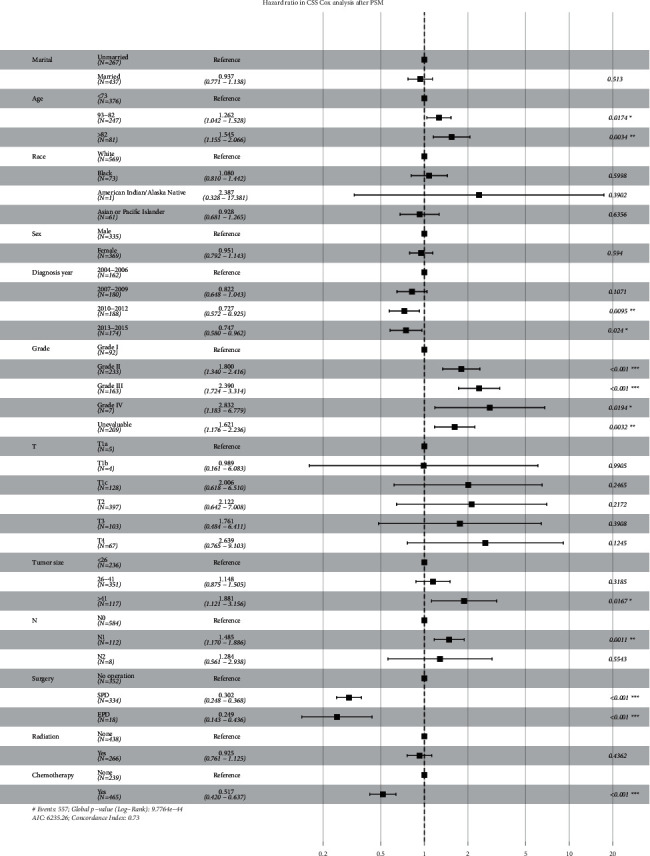
Hazard ratios in CSS (cancer-specific survival) Cox analysis after PSM (propensity score matching).

The Kaplan–Meier survival curves of OS and CSS before and after PSM are shown in Figures 1 and 2, respectively. The median follow-up time of OS in the nonsurgical group and the surgical group before PSM was 8.00 (4.00–14.00) and 20.00 (12.00–44.00), respectively. The figure in CSS was 8.00 (4.00–15.00) and 22.00 (12.00–53.00), respectively. The results showed that the median follow-up time of OS and CSS in the surgical group was significantly higher than those in the nonsurgical group (*P*=0.001), indicating that patients with pancreatic head adenocarcinoma aged 60 years or older can obtain a significant survival advantage from surgery. The median follow-up time of OS in the SPD and EPD groups before PSM was 20.00 (12.00–45.00) and 22.00 (10.00–38.00), *P*=0.636, respectively. The figure in CSS was 22.00 (12.00–53.00) and 22.00 (11.00–38.00), *P*=0.270, respectively. The results showed that there was no difference in the median follow-up time of OS and CSS between the SPD and EPD groups before PSM. The variable distribution of 704 cases (352 pairs) became more balanced after PSM. The median follow-up time of OS in the nonsurgical group and the surgical group before PSM was 9.00 (4.00–16.00) and 22.00 (12.00–62.00), respectively. The figure in CSS was 10.00 (4.00–17.00) and 25.00 (13.00–76.00), respectively. The survival curve of the nonsurgical group was lower than that of the surgical group in OS and CSS (*P* < 0.001). The median follow-up time of OS in the SPD and EPD groups after PSM was 23.00 (12.00–64.00) and 18.00 (12.00–44.00), *P*=0.415, respectively. The figure in CSS was 26.00 (13.00–76.00) and 18.00 (12.00–60.00), *P*=0.329, respectively. There was no difference in the median follow-up time of OS and CSS between the SPD and EPD groups after PSM. The multivariate CSS Cox regression analysis results of cohort 2 are displayed in the forest plot. As depicted in Figure 3, surgery is an independent risk factor. The HR of patients undergoing EPD surgery was 0.249 (0.143–0.436), and the HR in the SPD group was 0.302 (0.248–0.368). Although the HR value of EPD is lower, it shows that the patients can benefit more from EPD compared with the nonsurgical group. However, considering that there is no difference in survival data between the SPD and EPD groups, SPD is a more suitable surgical method for these patients.

## 4. Discussion

Pancreatic cancer is a highly malignant tumor, and its incidence increases by 1% per year [1]. The acceleration of social aging may lead to an increase in the morbidity. The detailed pathogenesis of pancreatic cancer is still unknown, but some factors have proven to be relevant significant independent risk factors, such as smoking, obesity, chronic pancreatitis, and diabetes [22]. Currently, R0 surgical resection is the only effective treatment for pancreatic cancer [15]. However, less than 20% of patients underwent surgery after initial diagnosis, and the invasive biological behavior of tumors leads to an advanced stage for most patients during the first diagnosis. Surgical resection in early-stage elderly patients was insufficient [16, 23]. Their age, underlying disease, and medical expenses may be key factors that affect the willingness of surgery for elderly patients. Pancreatic head cancer is the most common anatomical type, accounting for nearly 60–70% [3]. Tumor epidemiological characteristics and social aging make elderly patients account for larger proportions. It is still controversial whether they can benefit from more radical operations. Few studies are focusing on the optimization of surgical methods for these patients. Therefore, it is necessary to find a better surgical option for elderly patients with pancreatic head cancer.

The prognosis of patients who received surgical resection is significantly better than that of unresectable patients [15]. A systematic meta-analysis by Tan et al. demonstrated that elderly patients can receive surgery safely with acceptable risks by specialists in experienced centers and age should not be the only decisive factor in choosing patients for surgical treatment [24]. A population-based study in the Netherlands found the long-term survival rate of octogenarians who underwent operation is similar to that of the younger patients despite the high short-term mortality [25]. In our study, patients in the surgical group, including SPD and EPD, had better survival rates than those who did not receive surgery. This conclusion is the same as the results of some previous studies [26].

SPD is classical surgery for pancreatic head cancer, and its surgical resection rate was nearly 15%. For those patients who received the operation, the median survival time was 12–16 months, and the 5-year survival rate was only 5% [27]. Owing to the poor postoperative prognosis after SPD surgery, some scholars proposed a radical operation method based on the SPD, namely EPD surgery [28]. Recently, it has become more common in clinical practice with the improvement of surgical techniques and perioperative management. In many medical centers, EPD increased the operation time and intraoperative blood loss, but it did not increase the incidence of postoperative complications and guaranteed the safety of surgery [29].

However, whether expanding the surgical coverage can improve the survival benefit is still controversial. Fabrice et al. [30] found that the postoperative complication and mortality rate after EPD were significantly higher than those in the SPD group, and extended resection is an independent risk factor for postoperative death. Werner et al. [31] demonstrated that the mortality and long-term survival after EPD have no statistical differences with SPD, and the complication rate in EPD was higher than SPD. There is little research aimed at nonmetastatic pancreatic head cancer for the elderly.

In this study, accurate enrollment criteria are important prerequisites to explore the risk factors that affect the survival of elderly patients with nondistant metastatic pancreatic head adenocarcinoma. The eligible patients who have detailed clinical variables were collected from the SEER database, which makes the clinical analysis more convincing and rigorous. Independent risk factors were concluded from univariate and multivariate Cox models. PSM analysis was applied to adjust confounding variables and reduce selection bias, which makes the systematic differences of results can be attributed to treatment methods. The average survival time and median survival time are indicators commonly used in cancer research [32]. We also use this variable to compare the survival difference in this study and found that there was no survival difference between the EPD and SPD groups before and after PSM, and the surgical expansion did not bring significant survival benefits. The increase in surgical time, intraoperative blood loss, and postoperative complications also reduced postoperative quality of life, which has been demonstrated by many clinical studies. Hence, SPD is a better choice for nondistant metastatic pancreatic head adenocarcinoma patients compared with EPD. Except for surgery, the molecular type currently has guidance for clinical treatment. Immunotype pancreatic cancer has benefited from immunotherapy due to the expression of tumor-specific antigens and related immunocytes [33]. In addition, chemotherapy has improved the status in the treatment with the emergence of new chemotherapy strategies (albumin-binding paclitaxel, gemcitabine, and Folfirinox) [34, 35]. Preoperative new adjuvant chemotherapy is also an option. It is possible to screen the beneficial patients, increase the R0 resection rate and surgical efficacy, and promote survival benefits. As far as we know, this is the first study of nonmetastatic pancreatic head adenocarcinoma based on large sample size. This may provide surgeons with some help when making a better choice for these patients.

There are also some limitations to our study. Firstly, we conducted the PSM analysis to reduce the influence of confounding factors and eliminate selection bias. The smaller sample and loss of representativeness after PSM are still inevitable. The survival benefit of EPD needs to be verified by more prospective randomized clinical trials. Secondly, many variables in the SEER database do not exist or are incomplete, but they are closely related to the survival rate, such as R0 or R1 resection, information about postoperative radiotherapy, chemotherapy, and postoperative complications, etc. Thirdly, the SEER data were collected from multiple centers rather than a single hospital, and the treatment level of different hospitals has a great impact on the prognosis of patients. Given higher incidence of postoperative complications, EPD is recommended by the surgeon with rich experience in the large pancreatic cancer centers.

## 5. Conclusion

In short, surgery is an independent prognostic factor of elderly patients with nondistant metastatic pancreatic head adenocarcinoma, which has a better survival outcome than nonsurgical options. Age should not be the only decisive factor in choosing patients for surgical treatment. In the selection of specific surgical methods, SPD may provide these patients a better survival advantage compared with other surgical methods. We believe that SPD is a reasonable treatment option for patients with nondistant metastatic pancreatic head adenocarcinoma. Of course, this may require more randomized controlled clinical trials for further verification.

## Figures and Tables

**Table 1:** Baseline characteristics of all cases.

Terms	No. of patients (%)		*P*-value
	Nonsurgery (*N* = 2333)	Surgery (*N* = 984)	
Marital status (%)			<0.001
Unmarried	1037 (44.4)	336 (34.5)	
Married	1296 (55.6)	648 (65.9)	
Race (%)			<0.001
White	1839 (78.8)	842 (85.6)	
Black	274 (11.7)	75 (7.6)	
American Indian/Alaska Native	18 (0.8)	1 (0.1)	
Asian or Pacific Islander	202 (8.7)	66 (6.7)	
Sex (%)			0.016
Male	1056 (45.3)	491 (49.9)	
Female	1277 (54.7)	493 (50.1)	
Grade (%)			<0.001
Well differentiated; grade I	84 (3.6)	71 (7.2)	
Moderately differentiated; grade II	193 (8.3)	417 (43.7)	
Poorly differentiated; grade III	185 (7.9)	356 (35.6)	
Undifferentiated; grade IV	13 (0.6)	10 (1.0)	
Unevaluated	1858 (79.6)	130 (13.2)	
T (%)			<0.001
T1a	3 (0.1)	10 (1.0)	
T1b	9 (0.4)	11 (1.1)	
T1c	231 (9.9)	156 (15.9)	
T2	1197 (51.3)	604 (61.4)	
T3	408 (17.5)	167 (17.0)	
T4	485 (20.8)	36 (3.6)	
N (%)			<0.001
N0	2259 (96.8)	368 (37.4)	
N1	72 (3.1)	364 (37.0)	
N2	2 (0.1)	252 (25.6)	
Radiation (%)			<0.001
None	1556 (66.7)	580 (58.9)	
Yes	777 (33.3)	404 (41.1)	
Chemotherapy (%)			<0.001
None	1010 (43.3)	267 (27.1)	
Yes	1323 (56.7)	717 (72.9)	
Age, years			<0.001
Mean (SD)	75.55 (8.70)	70.61 (6.67)	
Diagnosis year			<0.001
2004–2006	380 (16.3)	208 (21.1)	
2007–2009	490 (21.0)	261 (26.5)	
2010–2012	687 (29.4)	260 (26.4)	
2013–2015	776 (33.3)	255 (26.0)	
Tumor size			<0.001
Mean (SD)	35.13 (17.79)	31.96 (13.87)	

**Table 2:** Baseline characteristics of propensity-score-matched cases.

Terms	No. of patients (%)		*P*-value
	Nonsurgery (*N* = 352)	Surgery (*N* = 352)	
Marital status (%)			0.277
Unmarried	141 (40.1)	126 (35.8)	
Married	211 (59.9)	226 (64.2)	
Race (%)			0.140
White	276 (78.4)	293 (83.2)	
Black	45 (12.8)	28 (8.0)	
American Indian/Alaska Native	0 (0.0)	1 (0.3)	
Asian or Pacific Islander	31 (8.8)	30 (8.5)	
Sex (%)			0.651
Male	164 (46.6)	171 (48.6)	
Female	188 (53.4)	181 (51.4)	
Grade (%)			<0.001
Well differentiated; grade I	75 (21.3)	17 (4.8)	
Moderately differentiated; grade II	115 (32.7)	118 (33.5)	
Poorly differentiated; grade III	43 (12.1)	120 (34.1)	
Undifferentiated; grade IV	3 (0.9)	4 (1.1)	
Unevaluated	116 (33.0)	93 (26.5)	
T (%)			0.320
T1a	3 (0.9)	2 (0.6)	
T1b	1 (0.3)	3 (0.9)	
T1c	62 (17.6)	66 (18.8)	
T2	199 (56.5)	198 (56.2)	
T3	46 (13.1)	57 (16.1)	
T4	41 (11.6)	26 (7.4)	
N (%)			0.312
N0	291 (82.7)	293 (83.2)	
N1	59 (16.8)	53 (15.1)	
N2	2 (0.5)	6 (1.7)	
Radiation (%)			0.312
None	226 (64.2)	212 (60.2)	
Yes	126 (35.8)	140 (39.8)	
Chemotherapy (%)			0.203
None	128 (36.4)	111 (31.5)	
Yes	224 (63.6)	241 (68.5)	
Age, years			<0.001
<73	179 (50.9)	197 (56.0)	
73–82	114 (32.3)	133 (37.8)	
>82	59 (16.8)	22 (6.2)	
Diagnosis year			0.038
2004–2006	96 (27.3)	66 (18.8)	
2007–2009	85 (24.1)	95 (27.0)	
2010–2012	94 (26.7)	94 (26.7)	
2013–2015	77 (21.9)	97 (27.5)	
Tumor size (mm)			0.017
<26	103 (29.3)	133 (37.8)	
26–41	194 (55.1)	157 (44.6)	
>41	55 (15.6)	62 (17.6)	

**Table 3:** Univariate and multivariate analysis of CSS Cox model before PSM.

Term	Univariate Cox analysis			Multivariate Cox analysis		
	HR	(95% CI)	*P*-value	HR	(95% CI)	*P*-value
Marital status	0.806	0.748–0.868	<0.001	0.943	0.871–1.022	0.152
Age (years)	1.456	1.385–1.530	<0.001	1.148	1.087–1.211	<0.001
Race	1.029	0.986–1.073	0.189	1.014	0.971–1.059	0.529
Sex	1.055	0.980–1.135	0.154	0.992	0.918–1.072	0.841
Diagnosis year	0.996	0.963–1.031	0.830	0.979	0.945–1.014	0.236
Grade	1.223	1.190–1.258	<0.001	1.026	0.993–1.059	0.121
T stage	1.186	1.142–1.232	<0.001	1.046	0.994–1.100	0.082
Tumor size	1.264	1.200–1.332	<0.001	1.216	1.138–1.299	<0.001
N stage	0.731	0.686–0.779	<0.001	1.281	1.180–1.390	<0.001
Surgery	0.351	0.321–0.382	<0.001	0.338	0.296–0.386	<0.001
Radiation	0.700	0.648–0.755	<0.001	0.863	0.794–0.939	0.001
Chemotherapy	0.495	0.459–0.534	<0.001	0.555	0.508–0.607	<0.001

Abbreviations: HR, hazard ratio; CI, confidence interval.

## Data Availability

All data were collected from the SEER database, which can be found on the official website: https://seer.cancer.gov/data/.

## Supplementary Materials

Supplementary Materials Supplementary information for this article can be found online. Supplementary Figure 1. The optimal cutoff values of age and tumor size were analyzed by X-tile software. The optimal cutoff values for age were 73 and 82 years; and the optimal cutoff values of tumor size were 26 and 41 mm.
