# Supplementary figures and images for: Elderly Patients with Nondistant Metastatic Pancreatic Head Adenocarcinoma Cannot Benefit from More Radical Surgery
Source: Int J Endocrinol. 2022 Apr 18;2022:6469740. doi: 10.1155/2022/6469740 (PMC9038409; doi:10.1155/2022/6469740)

A

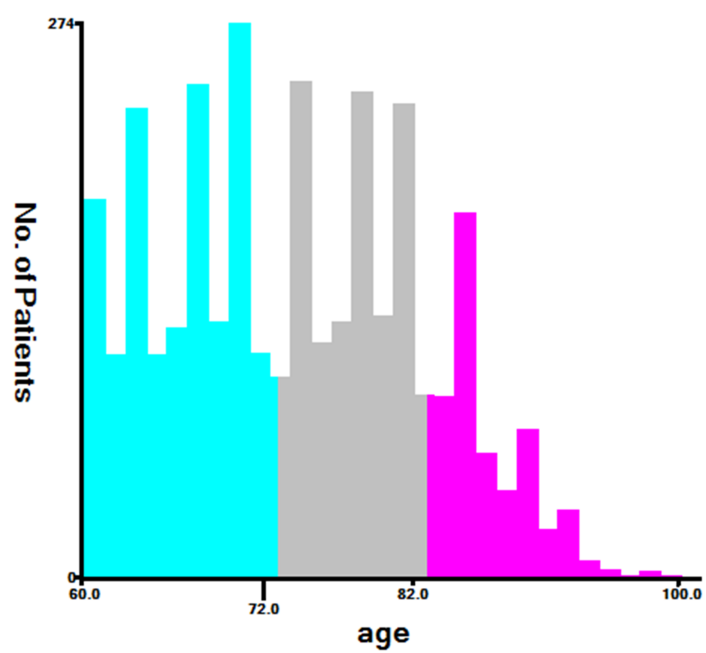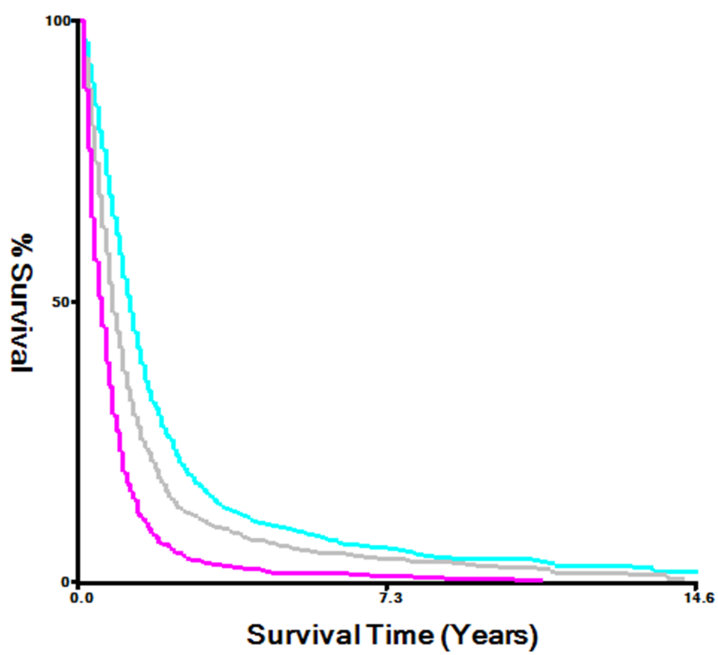

B

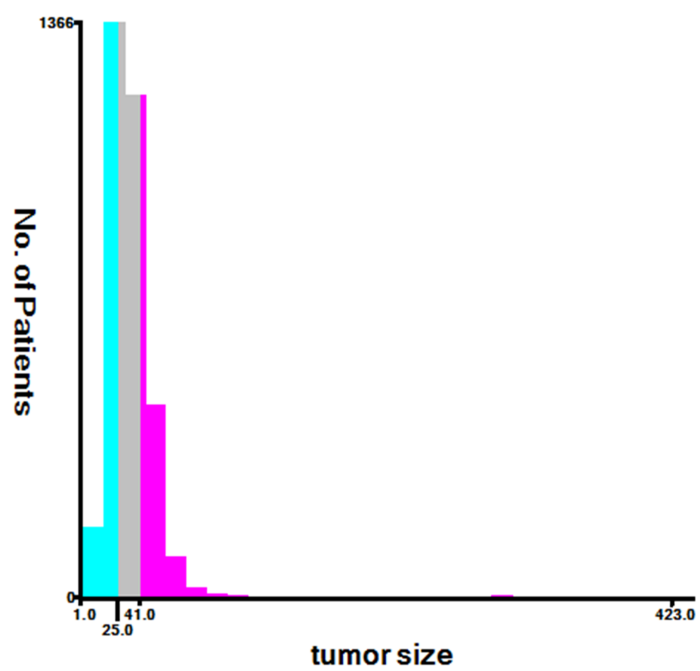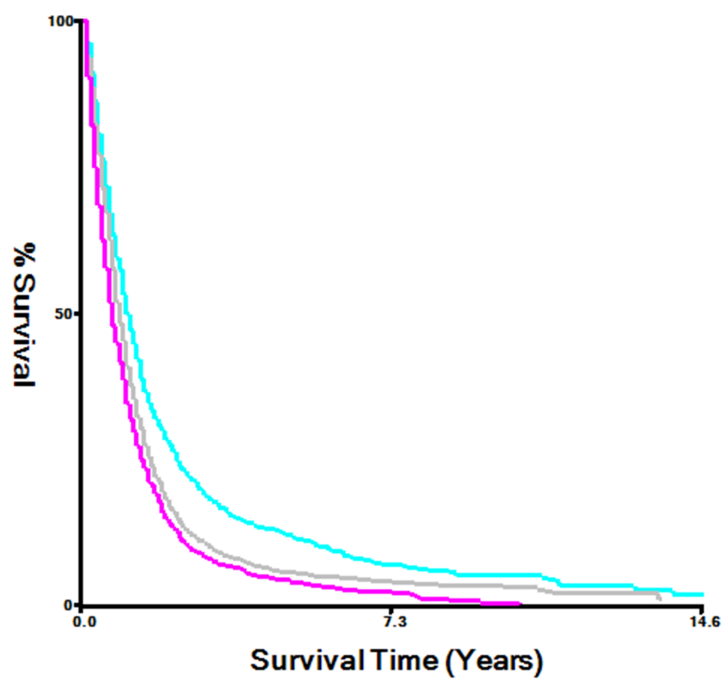

Supplement: Supplementary Materials — Supplementary information for this article can be found online. Supplementary Figure 1. The optimal cutoff values of age and tumor size were analyzed by X-tile software. The optimal cutoff values for age were 73 and 82 years; and the optimal cutoff values of tumor size were 26 and 41 mm. [file 6469740.f1.pdf]
